# Tortoise or Hare? The Associations between Physical Activity Volume and Intensity Distribution and the Risk of All-Cause Mortality: A Large Prospective Analysis of the UK Biobank

**DOI:** 10.3390/ijerph20146401

**Published:** 2023-07-19

**Authors:** Ruth Salway, Nicole Helene Augustin, Miranda Elaine Glynis Armstrong

**Affiliations:** 1 Centre for Exercise, Nutrition and Health Sciences, School for Policy Studies, University of Bristol, Bristol BS8 1TZ, UK; ruth.salway@bristol.ac.uk; 2 School of Mathematics, University of Edinburgh, Edinburgh EH9 3FD, UK; nicole.augustin@ed.ac.uk

**Keywords:** physical activity intensity distribution, all-cause mortality, prospective cohort study, methods development

## Abstract

Analysis methods to determine the optimal combination of volume and intensity of objectively measured physical activity (PA) with prospective outcomes are limited. Participants in UK Biobank were recruited in the UK between 2006 and 2010. We linked the questionnaire and accelerometer with all-cause mortality data from the NHS Information Centre and NHS Central Register up to April 2021. We developed a novel method, extending the penalized spline model of Augustin et al. to a smooth additive Cox model for survival data, and estimated the prospective relationship between intensity distribution and all-cause mortality, adjusting for the overall volume of PA. We followed 84,166 men and women (aged 40–69) for an average of 6.4 years (range 5.3–7.9), with an observed mortality rate of 22.2 deaths per 1000. Survival rates differed by PA volume quartile, with poorer outcomes for the lowest PA volumes. Participants with more sedentary to light intensity PA (<100 milligravities (mg)) and/or less vigorous intensity PA (>250 mg) than average for a given volume of PA, had higher mortality rates than vice versa. Approximate hazard ratios were 0.83 (95% credible interval [CI]: 0.79, 0.88) for an average-risk profile compared to a high-risk profile and 0.80 (95% CI: 0.74, 0.87) for a low-risk profile compared to an average-risk profile. A high- versus low-risk profile has the equivalent of 15 min more slow walking, but 10 min less moderate walking. At low PA volumes, increasing overall volume suggests the most benefit in reducing all-cause mortality risk. However, at higher overall volumes, substituting lighter with more vigorous intensity activity suggests greater benefit.

## 1. Introduction

Current physical activity (PA) guidelines are primarily based on studies assessing the associations between self-reported PA and health [1,2]. However, recent large-scale prospective studies that include accelerometry data, such as the UK Biobank [3], provide the opportunity to update the research base to underpin future iterations of PA guidelines using more objective measures. This also presents an opportunity for a step-change where the full spectrum of information available from accelerometry data is used rather than being limited to single-dimensional PA information [4].

The aerobic portion of current PA recommendations [2,5,6] is based around thresholds of duration and intensity: adults should do at least 150 min of moderate intensity activity or 75 min of vigorous intensity activity a week. Further, the aerobic section of the WHO guidelines suggests “an equivalent combination of moderate- and vigorous-intensity activity” (p. 2) [5], implying both total volume and intensity can contribute to health. However, there is insufficient evidence to make recommendations regarding the optimum balance between different combinations of volume and intensity of PA for health.

Current evidence suggests the dose-response relationship between PA and health outcomes is on a continuum, with no threshold [7,8,9]; hence, a single moderate-to-vigorous physical activity (MVPA) summary may discount the contribution of less intensive activity. Light-intensity PA can be beneficial, associated with reduced risk of all-cause mortality [10] and improvement in various health markers [11], even after adjusting for higher intensity activity [12]. Thus, more fully exploring the PA continuum will improve understanding of the complex nature of PA.

PA is a multidimensional behavior comprising not only intensity but also overall energy expenditure, duration, frequency and type of activity [13]. Different dimensions may be associated with different aspects of health. For example, the importance of PA volume versus intensity appears to differ for different outcomes and biomarkers [14,15,16]. Considering multiple dimensions of PA is a relatively new but promising area of research that takes advantage of the rich time series data produced by accelerometers. A new metric, the intensity gradient [17], avoids collinearity and so can be used alongside PA volume and was found to be independently associated with health outcomes in adults [17,18] and children [18,19]. Alternatively, compositional multiple linear regression avoids the problem of perfect multicollinearity of 24-h time budget data but relies on cut-points for sedentary, light, moderate, and vigorous activity [20]. Another option is to work with the intensity distribution in the form of a histogram, but this can be problematic due to the high collinearity of neighboring intensity bins. One approach [21] uses multivariate pattern analysis to deal with collinearity and has found that vigorous intensity activity was most strongly related to metabolic health in children. However, it uses proprietary software and cannot easily be extended for confounders. An alternative approach [22] uses a penalized spline to smooth the association across the histogram bins. They found a nonconstant, nonlinear relationship between fat mass and PA in adolescents, with lower fat mass associated with frequencies in the higher intensity bins. In neither case is it clear whether observed associations are due to more intensive, or higher overall PA volume.

There is a lack of prospective evidence on the relative importance of volume and intensity of PA, and it is unclear whether different PA profiles, in terms of volume and intensity, are equivalent or whether there are possible differences in their relationships with all-cause mortality. Therefore, exploring the multidimensional aspect of PA may help identify patterns of activity and better characterize the relationship between PA and all-cause mortality, ultimately informing behavior change. Our objectives were to extend the penalized spline model of Augustin et al. [22], which uses the histogram as a functional predictor, to a smooth additive Cox model, and to then estimate the prospective relationship between the intensity distribution and all-cause mortality, adjusting for the overall volume of PA, using data from the UK Biobank.

## 2. Materials and Methods

### 2.1. Design and Participants

The UK Biobank dataset is a large prospective study of over 500,000 adults aged between 40 and 69 years at recruitment, recruited at 22 assessment centers across England, Scotland, and Wales between 2006 and 2010. The UK Biobank received ethical approval from the North-West Multi-Centre Research Ethics Committee (REC reference: 11/NW/03820). All participants gave written informed consent prior to enrolment into the study [23], which was conducted in accordance with the principles of the Declaration of Helsinki. This paper analyses a subset of 103,107 participants supplied by the UK Biobank Resource (Application 30473), who provided accelerometer data between May 2013 and December 2015. We excluded: 6709 participants with prevalent cancers, i.e., with an existing diagnosis of any malignant neoplasm, except non-melanoma skin cancer (ICD 9 codes 140-208 excluding 173; ICD 10 codes with prefix C, excluding C44); 4920 participants with a history of coronary heart disease (ICD 9 codes 410-14; ICD 10 codes I20-25) and a further 1162 with a history of stroke (ICD 9 codes 430-38; ICD10 codes I60-69) prior to time of accelerometer data collection.

### 2.2. Measurements

#### 2.2.1. Outcome Data

Mortality data, provided by the NHS Information Centre and by the NHS Central Register, Scotland, are linked to UK Biobank participants on a quarterly basis. We calculated the time between baseline accelerometer data collection and date of death, or for censored cases, the time from baseline to the censoring date of 8 April 2021.

#### 2.2.2. Accelerometer Data

Participants wore an Axivity AX3 wrist-worn triaxial accelerometer (Axivity Ltd., Newcastle upon Tyne, United Kingdom) continuously on their dominant wrist for seven days. Accelerometer data were processed to produce data on acceleration (measured in milligravities) at five-second epochs, with non-wear time imputed based on similar time-of-day data; full details of data processing are available elsewhere [3]. Accelerometer data was considered valid if there were three or more days of data and data were present in each one-hour period of the 24-h cycle.

The mean acceleration (measured in milligravities [mg]) was calculated as the mean of all worn and imputed values over the seven-day period, as a proxy for PA volume. The distribution of time spent by an individual in different levels of PA acceleration [24] was captured via the empirical cumulative intensity distribution of five-second epochs. We converted these to relative frequencies for each interval, with accelerations > 500 mg combined into a single interval of 500–2000 mg to reduce the influence of outliers, as very few deaths (<5) occurred in participants with accelerations in this range. Accelerations of <3 mg were classified as sleep and excluded from analysis. For the purposes of this paper, sedentary refers to accelerations of <60 mg [25,26]. For clarity, in this paper, we use “PA volume” to refer to the mean acceleration and “intensity distribution” for the acceleration distribution excluding sleep.

#### 2.2.3. Other Covariates

Sex was recorded for all participants at recruitment. Age was calculated at the date of the accelerometer data collection. Confounders were measured at recruitment into the UK Biobank study, as there were too few measurements available at T1 (i.e., those measurements taken closest to the accelerometry readings) that also had accelerometer data. We included two measures of socioeconomic position: highest educational qualification and the Townsend Index of Deprivation [27] based on the participant’s postcode immediately prior to recruitment.

Participants reported their health status and any long-standing illness, disability, or infirmity. Participants were asked whether they smoked and how many alcoholic drinks they consumed per week. Body mass index (BMI) was calculated from height and weight measurements taken at the initial Assessment Centre visit and categorized based on WHO cut-offs [28]. As the BMI measurement preceded the accelerometer measurement, we treated it as a confounder, although, in general, the relationship may be bidirectional [29]. Please see Supplement S1 for further details on covariates.

### 2.3. Statistical Analysis

We compared characteristics of the accelerometer sample to those without accelerometer data and reported descriptive summaries and the proportion of missing variables. Due to the complexity of the statistical model, we did not impute missing data and analyses are based on 84,166 complete cases, under the assumption of missing at random. To investigate the extent to which these values change over time, we calculated the percentage of participants who reported the same value at recruitment and T1 for the subsample of repeated measurements.

We investigated the association between accelerometer-measured PA (both PA volume and intensity distribution) and all-cause mortality via a smooth additive Cox model for survival data based on time from accelerometer measurement. We used a generalized additive model (GAM) with a functional predictor following the approach of Augustin et al. [22], which modeled the all-cause mortality rate, via the hazard function, as a weighted average of relative frequencies for each interval of the intensity distribution histogram, where the weights are described by a smooth function (estimated by the model using a thin plate regression spline). Thus, some areas of the intensity distribution may contribute more to the overall mortality risk than others. The relationship between PA volume and all-cause mortality was modeled as a smooth function, using a thin plate regression spline smoother [30,31]. As the PA volume is a function of the intensity distribution (because mean intensity can be estimated from the histogram [22]), there is a close relationship between the two. To estimate associations with PA volume and intensity distribution separately, we removed the linear function (relating to mean intensity) from the basis of the smoother. Thus, the smooth term for the intensity distribution describes any additional association after adjusting for differences in PA volume. We fitted an unadjusted model with PA volume and intensity distribution only and an adjusted model, adjusting for sex, age, education, Townsend deprivation, self-reported poor health, long-term illness, current smoking, exceeding alcohol guidelines, and BMI category. We included the assessment center as a random effect term to account for possible clustering effects in the study design. Further technical details and sample R code are provided in the Technical Supplement S2.

The model was summarized by plotting the estimated smooth functions for the PA volume and intensity distribution and calculating hazard ratios (HRs). To describe the nonlinear association between PA volume and mortality risk, we estimated HRs for an increase of 1 mg in PA volume (roughly equivalent to replacing 10 min of sedentary time with 10 min of moderate walking [moderate activity] [25,26]) for different parts of the curve. Interpreting the intensity distribution smooth function is complex and depends on the comparison of different PA intensity distributions. We constructed approximate HRs and 95% credible intervals to compare high-risk, low-risk, and medium-risk intensity distributions (risk profiles) using a simulation-based approach and plotted HRs for different risk profiles by PA volume; full details are given in Supplement S1. To facilitate the interpretation of the high- and low-risk profiles, for each profile we calculated the average time spent at different PA volumes (sedentary: <60 mg; equivalent to slow walking: 60–125 mg; equivalent to moderate walking: 125–300 mg; equivalent to brisk walking or higher: 300+ mg) based on typical intensities [25,26].

This method ensured that the risk profiles represent realistic patterns of PA but should be interpreted with caution and used to indicate the order of magnitude of the potential risk associated with different distributional patterns, especially in comparison to other covariates in the model, rather than absolute estimates of risk.

To assess sensitivity to the COVID-19 pandemic, which occurred toward the end of the time frame of the included data, we repeated the analysis with an endpoint of the 1 March 2020. We also explored the sensitivity of the model to different choices for the spline parameters (both basis and penalty dimensions) and additionally considered nonlinear associations for age and separate intensity distributions by sex. All analyses were undertaken in R v4.2.0 (R Foundation for Statistical Computing, Vienna, Austria) using the mgcv package.

## 3. Results

The average follow-up time from accelerometer data collection to the end of the current analysis on 8 April 2021 was 6.4 years (range 5.3–7.9 years), with an observed mortality rate of 22.2 deaths per 1000. Table 1 summarizes the characteristics of the dataset and compares the analysis sample with the full Biobank study (excluding deaths prior to accelerometer data collection).

Participants in the analysis sample were less likely to have a longstanding illness, report poor health, smoke, or have obesity. This is reflected in a lower mortality rate. Rates of missing data were low, at 2% for longstanding illness and <1% for other covariates. Of the 84,166 cases with valid accelerometer data, 3% were omitted due to missing covariate data, resulting in 81,752 complete cases. On average, confounders were measured 5.7 years before accelerometer data collection. In the subsample of repeated measurements, over 80% reported the same value, with agreement rising to over 90% for smoking, education, and poor health (Supplement S1, Table S1).

**Figure 1:**
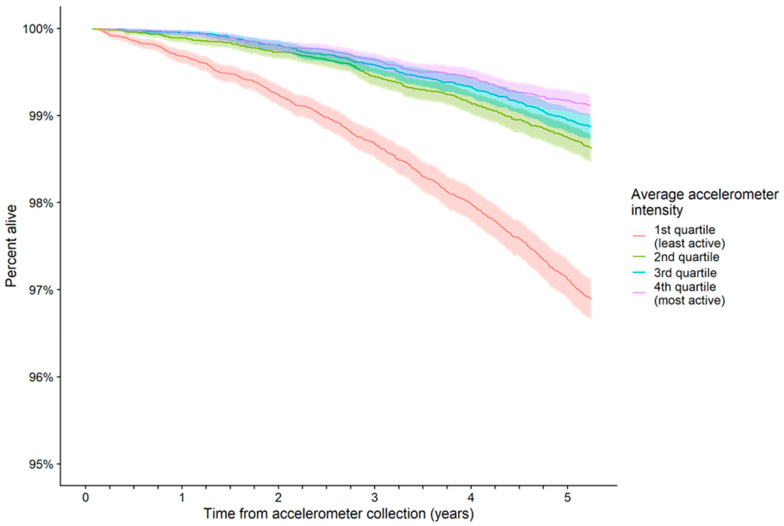
Kaplan-Meier survival curves and 95% confidence regions (shaded areas) by physical activity volume (average accelerometer intensity) quartiles: note y-axis does not start from zero. Kaplan-Meier log-rank test, *p* < 0.001.

Figure 1 shows Kaplan-Meier survival curves and 95% confidence bands by PA volume quartile; note the scale of the y-axis due to the short follow-up time. Survival rates differed by PA volume quartile, with poorer outcomes for the lowest PA volume. Supplement S1, Figure S1 shows the “average intensity distribution” of mean frequencies in each interval. Observed intensities ranged between 0 and 2000 mg with a median PA volume of 28.4 mg, and on average, only 0.2% of epochs recorded intensities above 500 mg.

**Figure 2:**
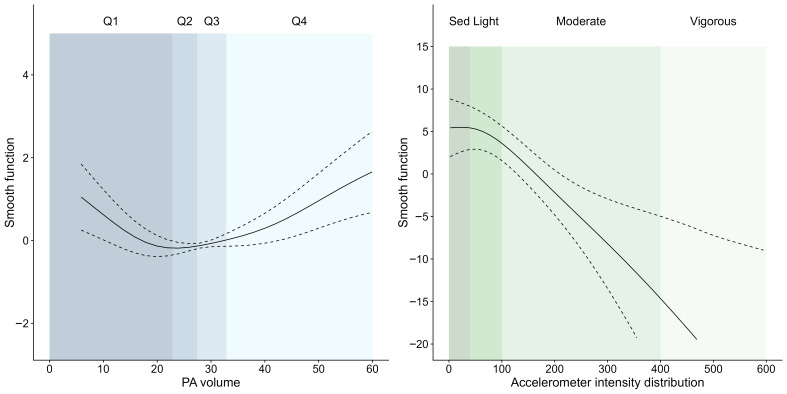
Associations between physical activity volume and intensity and all-cause mortality: physical activity volume (mean accelerometer intensity) (**left**) and histogram of intensity distribution (**right**). Solid line is the mean smooth function, with 95% confidence bands indicated by the dashed lines. PA = physical activity; Q = quartile; Sed = sedentary. The left panel shows the relationship with mean physical activity volume (mean accelerometer intensity, truncated to 99% of the data), with quartiles marked. The right panel shows the relationship with the distribution of intensity, with areas corresponding roughly to sedentary, light, moderate, and vigorous intensity activities.

HRs for the association between PA and all-cause mortality for the unadjusted and adjusted models are presented in Table 2. Figure 2 (left panel) shows a nonlinear association between PA volume and all-cause mortality risk, truncated to the region 0–60 mg, which includes over 99% of the data (full range in Supplement S1, Figure S2). The smooth function shows a high all-cause mortality risk for very low PA volume, with decreasing risk as PA volume increases (HR = 0.91; 95% CI: 0.87–0.96 per increase of 1 mg). The curve flattens between 20 mg and 30 mg, and then the risk increases until 80 mg (HR = 1.05; 95% CI: 1.01–1.09 per increase of 1 mg), where it decreases again, although with wide confidence bands (HR close to 1 per increase of 1 mg).

**Figure 3:**
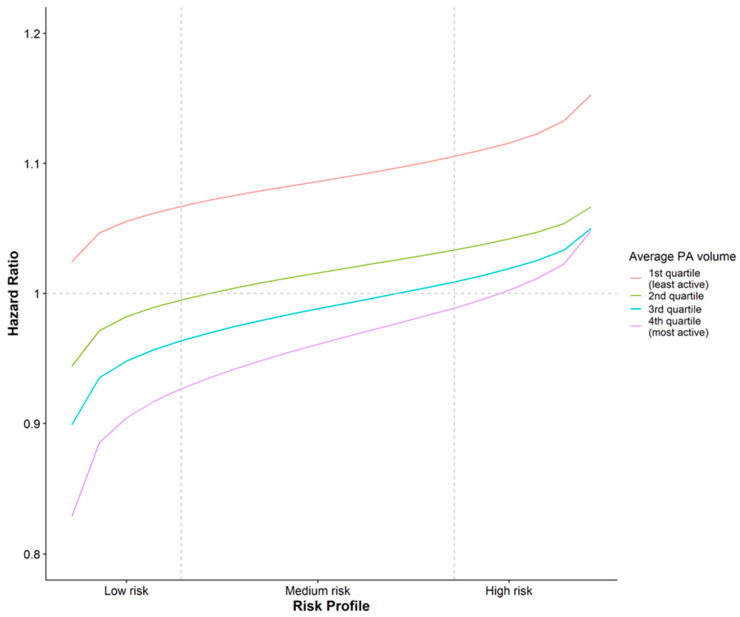
Hazard ratios by physical activity volume and risk profile. PA = physical activity. Hazard ratios are compared to a participant with an average PA volume and a medium-risk profile.

The smooth function of the intensity distribution (Figure 2, right panel) identifies areas of the intensity distribution associated with high and low all-cause mortality risk. The curve was highest between 3 and 100 mg (approximately sedentary to light intensity PA [32]), indicating an area of higher risk, and lowest above 250 mg (approximately MVPA [26]), indicating an area of lower risk. Thus, participants with more activity <100 mg and/or less over 250 mg than average for a given PA volume, had higher mortality rates than vice versa. Approximate HRs were 0.83 (95% credible interval [CI]: 0.79, 0.88) for an average-risk profile compared to a high-risk profile and 0.80 (95% CI: 0.74, 0.87) for a low-risk profile compared to an average-risk profile. Figure 3 compares the HRs for risk profiles by PA volume quartiles; all curves show increasing HRs as profiles change from low to higher risk, with curves for lower PA volume notably higher than those for high PA volume. To put these values in context, we translated milligravity intensities into equivalent typical mean walking paces (Table 3). A typical study participant engaged in the equivalent of ~2.3 h of slow walking per day in addition to a further hour of moderate walking and 7 min of brisk walking. Participants in the lowest quartile, on average, took the equivalent of 105 min of slow walking, 35 min of moderate walking, and 3 min brisk walking per day. For someone engaging in an average PA volume of 25 mg (Quartile 2), a high-risk profile would have the equivalent of ~15 min more slow walking and 10 min less moderate walking compared to an average risk profile and ~30 min more slow walking, but ~15 min less moderate walking and ~5 min less brisk walking, compared to a low-risk profile.

Age and sex were strongly related to all-cause mortality, with risk more than doubling for every increase in 10 years in age (HR = 2.57; 95% CI: 2.38, 2.78; Table 2), and men with around 65% higher risk than women (95% CI: 1.49, 1.83). Poor health or a longstanding illness were both associated with higher mortality (HR = 1.61; 95% CI:1.27, 2.03 and 1.39; 95% CI: 1.25, 1.53, respectively). Among lifestyle confounders, smoking had the strongest association (HR = 1.85; 95% CI: 1.60, 2.14) with a weaker association with obesity, compared to healthy weight (HR = 1.20; 95% CI: 1.05, 1.36). Associations with socioeconomic variables (deprivation and education) were weak (Table 2).

We reran the analysis using an endpoint of 1 March 2020 to exclude the COVID pandemic (Supplement S1, Table S2) and found that HRs were broadly similar. We explored more complex models but found no evidence that the association between age and mortality risk was nonlinear, or that the association with intensity distributions differed by sex (Supplement S1, Table S3).

## 4. Discussion

Both PA volume and intensity distribution were associated with all-cause mortality. Those with very low levels of PA volume were at higher risk of mortality, with the risk reducing by 9% for every 1 mg increase in PA volume; roughly equivalent to replacing 10 min of sedentary time with 10 min of brisk walking [25,26]. Conversely, the risk increased slightly at higher levels of PA volume, by 5% for every 1 mg increase in PA volume greater than 30 mg. Associations with intensity distribution were complex and exhibited high variability, making conclusions difficult despite the large sample sizes. However, we identified a high-risk intensity profile as one with relatively more sedentary to light intensity PA (<100 mg) and/or relatively less vigorous intensity PA (>250 mg), with a low-risk profile and vice versa.

Comparisons between high- and low-risk profiles should be treated with some caution. The HR for an average-risk intensity profile compared to a low-risk intensity profile was 1.20; a similar order of magnitude to the HR for obesity or a decrease in PA volume of 2 mg at very low PA volumes. This suggests both PA volume and PA intensity distribution are important and that the relative importance of the two may change as PA volumes increase. At higher levels of PA volume, moving from a high-risk profile to a lower-risk profile may offer more health benefits than increasing the PA volume. Those in the lowest 15% of PA volume, who currently do around 2 h or less of any intensity of PA (PA volume < 20 mg) per day should consider increasing their total PA volume. For example, swapping 20 min of sedentary time for 20 min of brisk walking per day (thereby increasing PA volume by 2 mg) was associated with a 20% lower all-cause mortality risk. Those who currently do a greater volume of PA should consider increasing intensity. For example, swapping 20–30 min of slow walking for ~15 min of faster walking, split between brisk and fast walking (hence moving from a high-risk to an average-risk profile), is associated with the same ~20% reduction in mortality risk.

The literature on the joint prospective association of PA volume and intensity together with all-cause mortality risk is limited. A systematic review and harmonized meta-analysis concluded that higher volumes of total PA, at any intensity, were associated with a reduced risk of premature mortality compared to low volumes of PA, with risks for high and very high PA volumes similar [9]. They found a nonlinear dose-response pattern for total PA volume (measured in counts per minute) very similar to ours, with initial decreasing risk followed by a shallower increase. However, unlike our approach, these models did not include a mutual adjustment for both volume and intensity. A systematic review and meta-analysis of light PA and cardiometabolic health and mortality [11] found that the effects reported for light PA were two to four times smaller than for the same amount of time spent in MVPA, which implies benefits of higher PA volume, irrespective of intensity. Comparisons of models for total PA count and MVPA have suggested that PA volume has stronger associations with insulin resistance [14] and cardiometabolic biomarkers [15] than MVPA, but as these two measures are highly correlated, it is difficult to compare the associations directly.

There are challenges in exploring intensity distributions. For example, mean intensity is a function of the intensity distribution, and there is a high correlation in the histogram between levels of similar intensities. The GAM model described in this paper has the benefit of dealing with both issues. However, a large amount of data is required, especially when looking at long-term mortality. Therefore, high-quality, large sample cohorts such as the UK Biobank are essential if we want to understand the nuances of PA. Even with this large dataset, confidence intervals were wide in areas of little data.

A clear strength of this study includes the novel analysis method developed. This allows for the prospective assessment of nonlinear associations of PA volume and PA intensity with mortality, enabling us to make more extensive use of accelerometer data. We were able to separate the PA volume from the intensity distribution so that the distribution captures differences in patterns rather than higher overall levels of PA, thereby allowing us to compare the relative contributions of PA volume and intensity.

This study is limited by the short follow-up time and few deaths in the sample. Further, it was not possible to look at specific causes of death, which would require more events or longer follow-up times. Those participants who completed the accelerometer data collection and were included in this analysis were healthier than Biobank participants as a whole, with a lower prevalence of lifestyle risk factors such as smoking, alcohol consumption, and obesity. It is, therefore, possible that they would be more likely to engage in physical activity. If this is the case, then it may mean we have underestimated the effects among those who are very inactive, although we note that adjusting for these factors in our analysis will reduce participation bias from this source. The UK Biobank consists primarily of white participants from less socioeconomically deprived areas, which may reduce generalizability. However, its large sample size and extensive exposure data allow valid estimates of the relationships between exposures and disease outcomes [33,34]. To maximize sample size, we used confounders measured 5–6 years before the accelerometer data, which could weaken associations if confounders change over time. However, our comparison suggests high consistency, with 82–97% reporting the same value at two time points. Finally, all covariates are measured at a single time point, so changes in PA, illness, and lifestyle factors will weaken the analysis and make it hard to spot associations. In particular, PA distributions may change with age.

## 5. Conclusions

Current physical activity (PA) guidelines are primarily based on self-reported measures of PA. Recent large cohort studies, such as the UK Biobank, with objective measures of PA [3] give scope for producing evidence for the relationship between objectively measured PA and health outcomes. However, analysis methods are limited in their ability to determine the optimal combination of volume and intensity of PA using prospective outcome data. We have developed a new analysis method that overcomes the limitations of currently available analysis methods, taking advantage of the extensive data available in large-scale cohorts such as UK Biobank. We have successfully implemented this method to improve our understanding of the intricate relationship between PA intensity distribution and overall PA volume with all-cause mortality. This new method will allow for the examination of these highly correlated PA exposures with a variety of other health outcomes in the future.

Overall, our results showed that the distribution of PA was an important factor in all-cause mortality, especially for higher volumes of PA. At low PA volumes, increasing overall PA volume suggests the most benefit in reducing all-cause mortality risk. However, at higher overall volumes, substituting lighter PA with more vigorous intensity PA suggests greater benefit.

## Figures and Tables

**Table 1:** Data characteristics of the sample and missing data.

	Without Accelerometer Data	With Valid Accelerometer Data	
	Mean (sd) or %	Mean (sd) or %	Missing (%)
Total	477,806	84,166	
All-cause mortality rate (per 1000)	61.7 (240.6)	22.2 (147.4)	0
Years from Biobank baseline to death	8.8 (2.4)	9.6 (1.9)	0
All-cause pre-COVID ^a^ mortality rate (per 1000)	49.3 (216.4)	16.3 (126.5)	0
Accelerometer PA volume (mg) ^b^		28.4 (8.3)	0
Sex (% female)	54%	58%	0
Age at Biobank baseline	56.5 (8.2)	55.6 (7.8)	0
Age at accelerometer collection		61.8 (7.8)	0
Education: degree or higher	36%	49%	376 (0.4%)
Townsend index	−1.21 (3.14)	−1.73 (2.82)	97 (0.1%)
Self-reported health: poor	5%	2%	173 (0.2%)
Longstanding illness	33%	26%	1559 (2.0%)
Current smoker	11%	7%	223 (0.3%)
Exceeds alcohol guidelines	33%	32%	71 (0.1%)
BMI category			172 (0.2%)
Underweight/Healthy weight	32%	41%	
Overweight	43%	41%	
Obese	26%	19%	

sd = standard deviation; PA = physical activity; BMI = body mass index; ^a^ excludes all deaths after 1 March 2020; ^b^ refers to the overall volume of PA (accelerometer mean intensity).

**Table 2:** Hazard ratios for the association between physical activity volume and intensity histogram and mortality (n = 81,752).

	Unadjusted		Adjusted ^d^	
	HR	95% CI	HR	95% CI
Adjustment variables				
Male (versus female)			1.65	(1.49, 1.83)
Age (10-year increase)			2.57	(2.38, 2.78)
Degree-educated			0.89	(0.81, 0.97)
Townsend index (1 sd ^a^ increase)			1.02	(1.00, 1.04)
Self-reported poor health			1.61	(1.27, 2.03)
Longstanding illness			1.39	(1.25, 1.53)
Current smoker			1.85	(1.60, 2.14)
Exceeds alcohol guidelines			1.09	(0.98, 1.20)
Overweight ^b^			0.93	(0.83, 1.04)
Obese ^b^			1.20	(1.05. 1.36)
Mean PA volume (increase of 1 mg) ^c^				
0–20 mg	0.92	(0.87, 0.97)	0.91	(0.87, 0.96)
20–30 mg	1.02	(0.98, 1.06)	1.01	(0.98, 1.04)
30–80 mg	1.09	(1.05, 1.13)	1.05	(1.01, 1.09)
80 mg+	1.00	(0.90, 1.11)	0.98	(0.88, 1.09)
PA intensity histogram:				
Move from high risk to average risk	0.69	(0.65, 0.73)	0.83	(0.79, 0.88)
Move from average risk to low risk	0.67	(0.62, 0.72)	0.80	(0.74, 0.87)
Move from high risk to low risk	0.46	(0.41, 0.52)	0.67	(0.58, 0.76)

HR = hazard ratio; CI = confidence interval; sd = standard deviation; PA = physical activity; mg = milligravities; ^a^ Townsend standard deviation = 2.8; ^b^ Compared to “not overweight or obese”; ^c^ Refers to the mean physical activity volume (accelerometer mean intensity). An increase of 1 mg is roughly equivalent to replacing 10 min of sedentary time with 10 min of moderate walking; ^d^ Adjusted for sex, age, education, Townsend deprivation, self-reported poor health, long-term illness, current smoking, exceeding alcohol guidelines, and BMI category.

**Table 3:** Median time spent in various milligravity ranges for different intensity risk profiles within physical activity volume quartiles ^a^.

	Sleep (h)	Sedentary (h)	Equivalent to Slow Walking (~3 km/h) (min)	Equivalent to Moderate Walking (~5 km/h) (min)	Equivalent to Brisk Walking or Higher (~6.5 km/h) ^b^ (min)
Intensity (mg) ^c^	<3	3- < 60	60- < 125	125- < 300	300+
Overall average	7.2	13.2	140	62	7
Quartile 1: (mean PA volume =19.2 mg)					
High-risk profile	7.3	14.3	109	26	1
Average risk profile	7.9	13.7	105	35	3
Low-risk profile	8.5	13.1	94	42	4
Quartile 2: (mean PA volume =25.2 mg)					
High-risk profile	6.7	14.0	150	45	3
Average risk profile	7.3	13.4	135	55	6
Low-risk profile	8.0	12.9	117	60	9
Quartile 3: (mean PA volume =30.0 mg)					
High-risk profile	6.3	13.5	179	63	4
Average risk profile	7.0	13.1	156	73	9
Low-risk profile	7.7	12.6	130	76	14
Quartile 4: (mean PA volume =39.4 mg)					
High-risk profile	5.9	12.7	210	96	10
Average risk profile	6.6	12.4	177	102	15
Low-risk profile	7.2	12.3	144	94	24

PA = physical activity; mg = milligravities; h = hours; min = min; km = kilometers; ^a^ For ease of interpretation we have presented intensity data according to equivalent walking speeds, although in practice, the intensity could have been accumulated through a variety of activity types; ^b^ The category “Equivalent to fast walk or higher” is nearly all <600 mg with no time spent at intensities higher than 750 mg (equivalent to a slow run); ^c^ Typical intensities based on definitions given in Hildebrand et al. (2014) [26] and Esliger et al. 2011 [25].

## Data Availability

UK Biobank is an open access resource. Bona fide researchers can apply to use the UK Biobank data set by registering and applying at http://www.ukbiobank.ac.uk/register-apply/. The statistical code used for this manuscript is available to other researchers in the supplement of this manuscript.

## Supplementary Materials

The following supporting information can be downloaded at: https://www.mdpi.com/article/10.3390/ijerph20146401/s1, S1: Supplement S1; S2: Technical Supplement; Figure S1: Mean histogram of accelerometer intensity: bars represent the average density in each bin across all participants, Figure S2: Associations between physical activity volume and mortality: full range of data for mean accelerometer intensity; Table S1: Change in confounders between recruitment and T1 (includes those not in accelerometer subsample) N = 20,264, Table S2: Hazard ratios for the association between physical activity volume and intensity histogram and mortality (excluding COVID pandemic), Table S3: Model fit for more complex models. References [35,36,37] are cited in the Supplement S1. References [38,39,40,41] are cited in the Supplement S2.

## References

[1] US Department of Health and Human Services (2018). Physical Activity Guidelines Advisory Committee Scientific Report.

[2] UK Chief Medical Officers (2019). UK Chief Medical Officers’ Physical Activity Guidelines.

[3] Doherty A., Jackson D., Hammerla N., Plötz T., Olivier P., Granat M.H., White T., Van Hees V.T., Trenell M.I., Owen C.G. (2017). Large scale population assessment of physical activity using wrist worn accelerometers: The UK Biobank Study. PLoS ONE.

[4] Troiano R.P., McClain J.J., Brychta R.J., Chen K.Y. (2014). Evolution of accelerometer methods for physical activity research. Br. J. Sports Med..

[5] World Health Organization (2020). WHO Guidelines on Physical Activity and Sedentary Behaviour.

[6] Piercy K.L., Troiano R.P., Ballard R.M., Calrson S.A., Fulton J.E., Galuska D.A., George S.M., Olson R.D. (2020). The Physical Activity Guidelines for Americans. JAMA.

[7] Kyu H.H., Bachman V.F., Alexander L.T., Mumford J.E., Afshin A., Estep K., Veerman J.L., Delwiche K., Iannarone M.L., Moyer M.L. (2016). Physical activity and risk of breast cancer, colon cancer, diabetes, ischemic heart disease, and ischemic stroke events: Systematic review and dose-response meta-analysis for the Global Burden of Disease Study 2013. BMJ.

[8] Arem H., Moore S.C., Patel A., Hartge P., Berrington de Gonzalez A., Visvanathan K., Campbell P.T., Freedman M., Weiderpass E., Adami H.O. (2015). Leisure time physical activity and mortality: A detailed pooled analysis of the dose-response relationship. JAMA Intern. Med..

[9] Ekelund U., Tarp J., Steene-Johannessen J., Hansen B.H., Jefferis B., Fagerland M.W., Whincup P., Diaz K.M., Hooker S.P., Chernofsky A. (2019). Dose-response associations between accelerometry measured physical activity and sedentary time and all cause mortality: Systematic review and harmonised meta-analysis. BMJ.

[10] Loprinzi P.D. (2017). Light-Intensity Physical Activity and All-Cause Mortality. Am. J. Health Promot..

[11] Chastin S.F.M., De Craemer M., De Cocker K., Powell L., Van Cauwenberg J., Dall P., Hamer M., Stamatakis E. (2019). How does light-intensity physical activity associate with adult cardiometabolic health and mortality? Systematic review with meta-analysis of experimental and observational studies. Br. J. Sports Med..

[12] Amagasa S., Machida M., Fukushima N., Kikuchi H., Takamiya T., Odagiri Y., Inoue S. (2018). Is objectively measured light-intensity physical activity associated with health outcomes after adjustment for moderate-to-vigorous physical activity in adults? A systematic review. Int. J. Behav. Nutr. Phys. Act..

[13] Thompson D., Batterham A.M. (2013). Towards integrated physical activity profiling. PLoS ONE.

[14] Boyer W.R., Wolff-Hughes D.L., Bassett D.R., Churilla J.R., Fitzhugh E.C. (2016). Accelerometer-Derived Total Activity Counts, Bouted Minutes of Moderate to Vigorous Activity, and Insulin Resistance: NHANES 2003–2006. Prev. Chronic Dis..

[15] Wolff-Hughes D.L., Fitzhugh E.C., Bassett D.R., Churilla J.R. (2015). Total Activity Counts and Bouted Minutes of Moderate-To-Vigorous Physical Activity: Relationships With Cardiometabolic Biomarkers Using 2003–2006 NHANES. J. Phys. Act. Health.

[16] Wu F., Wills K., Laslett L.L., Oldenburg B., Jones G., Winzenberg T. (2017). Moderate-to-Vigorous Physical Activity But Not Sedentary Time Is Associated With Musculoskeletal Health Outcomes in a Cohort of Australian Middle-Aged Women. J. Bone Min. Res..

[17] Rowlands A.V., Edwardson C.L., Davies M.J., Khunti K., Harrington D.M., Yates T. (2018). Beyond Cut Points: Accelerometer Metrics that Capture the Physical Activity Profile. Med. Sci. Sports Exerc..

[18] Rowlands A.V., Fairclough S.J., Yates T., Edwardson C.L., Davies M., Munir F., Khunti K., Stiles V.H. (2019). Activity Intensity, Volume, and Norms: Utility and Interpretation of Accelerometer Metrics. Med. Sci. Sports Exerc..

[19] Buchan D.S., McLellan G., Donnelly S., Arthur R. (2019). The use of the intensity gradient and average acceleration metrics to explore associations with BMI z-score in children. J. Sports Sci..

[20] Dumuid D., Stanford T.E., Martin-Fernández J.-A., Pedišić Ž., Maher C.A., Lewis L.K., Hron K., Katzmarzyk P.T., Chaput J.-P., Fogelholm M. (2018). Compositional data analysis for physical activity, sedentary time and sleep research. Stat. Methods Med. Res..

[21] Aadland E., Kvalheim O.M., Anderssen S.A., Resaland G.K., Andersen L.B. (2018). The multivariate physical activity signature associated with metabolic health in children. Int. J. Behav. Nutr. Phys. Act..

[22] Augustin N.H., Mattocks C., Faraway J.J., Greven S., Ness A.R. (2017). Modelling a response as a function of high-frequency count data: The association between physical activity and fat mass. Stat. Methods Med. Res..

[23] Sudlow C., Gallacher J., Allen N., Beral V., Burton P., Danesh J., Downey P., Elliott P., Green J., Landray M. (2015). UK biobank: An open access resource for identifying the causes of a wide range of complex diseases of middle and old age. PLoS Med..

[24] Hammerla N.Y., Kirkham R., Andras P., Ploetz T. (2013). On Preserving Statistical Characteristics of Accelerometry Data Using Their Empirical Cumulative Distribution. Proceedings of the 2013 International Symposium on Wearable Computers.

[25] Esliger D.W., Rowlands A.V., Hurst T.L., Catt M., Murray P., Eston R.G. (2011). Validation of the GENEA Accelerometer. Med. Sci. Sports Exerc..

[26] Hildebrand M., VT V.H., Hansen B.H., Ekelund U. (2014). Age group comparability of raw accelerometer output from wrist-and hip-worn monitors. Med. Sci. Sports Exerc..

[27] Townsend P., Phillimore P., Beattie A. (1988). Health and Deprivation: Inequality and the North.

[28] World Health Organiszation (2015). Obesity and Overweight.

[29] Pedisic Z., Grunseit A., Ding D., Chau J.Y., Banks E., Stamatakis E., Jalaludin B.B., Bauman A.E. (2014). High sitting time or obesity: Which came first? Bidirectional association in a longitudinal study of 31,787 Australian adults. Obesity.

[30] Wood S. (2006). Generalized Additive Models: An Introduction with R.

[31] Wood S.N. (2003). Thin plate regression splines. J. R. Stat. Soc. Ser. B (Stat. Methodol.).

[32] Rowlands A.V., Mirkes E.M., Yates T., Clemes S., Davies M., Khunti K., Edwardson C.L. (2018). Accelerometer-assessed physical activity in epidemiology: Are monitors equivalent?. Med. Sci. Sports Exerc..

[33] Batty G.D., Gale C.R., Kivimäki M., Deary I.J., Bell S. (2020). Comparison of risk factor associations in UK Biobank against representative, general population based studies with conventional response rates: Prospective cohort study and individual participant meta-analysis. BMJ.

[34] Fry A., Littlejohns T.J., Sudlow C., Doherty N., Adamska L., Sprosen T., Collins R., Allen N.E. (2017). Comparison of sociodemographic and health-related characteristics of UK Biobank participants with those of the general population. Am. J. Epidemiol..

[35] Galobardes B., Shaw M., Lawlor D.A., Lynch J.W., Davey Smith G. (2006). Indicators of socioeconomic position (part 1). J. Epidemiol. Community Health.

[36] Galobardes B., Shaw M., Lawlor D.A., Lynch J.W., Davey Smith G. (2006). Indicators of socioeconomic position (part 2). J. Epidemiol. Community Health.

[37] (2016). UK Chief Medical Officers. UK Chief Medical Officers’ Low Risk Drinking Guidelines.

[38] Cox D.R. (1972). Regression models and life-tables. J. R. Stat. Soc. Ser. B (Methodol.).

[39] Hastie T., Tibshirani R. (1987). Generalized additive models: Some applications. J. Am. Stat. Assoc..

[40] Wood S., Pya N., Säfken B. (2016). Smoothing parameter and model selection for general smooth models (with discussion). J. Am. Stat. Assoc..

[41] Wood S. (2017). Generalized Additive Models: An Introduction with R.

