# Supplementary material for: Tortoise or Hare? The Associations between Physical Activity Volume and Intensity Distribution and the Risk of All-Cause Mortality: A Large Prospective Analysis of the UK Biobank
Source: Int J Environ Res Public Health. 2023 Jul 19;20(14):6401. doi: 10.3390/ijerph20146401 (PMC10378963; doi:10.3390/ijerph20146401)
Supplement: Supplementary file 1 [file ijerph-20-06401-s001.zip › Supplement S1.pdf]

Supplement S1: Supplementary Methods, Tables and Figures

Supplementary Methods

Further Details of other covariates

We included two measures of socio-economic position as different measures capture different, often related, aspects [35,36]. Participants were asked their highest educational qualification, which was recoded as ‘University degree (or equivalent) and higher’ versus ‘No university degree (or equivalent)’. In addition, we included the Townsend Index of Deprivation [27] based on the participant’s postcode immediately prior to recruitment. A higher score indicates a greater degree of deprivation.

Participants were asked to report their health on a scale of ‘Excellent’, ‘Good’, ‘Fair’ and ‘Poor’. We created an indicator variable of self-reported poor health based on those who responded ‘Poor’ to this question. In addition, all participants were asked if they had a long-standing illness, disability, or infirmity (reported as Yes or No).

Participants were asked about lifestyle factors including whether they smoked (coded as current smoker versus past or never smoker), and how many alcoholic drinks of different types (beer, red wine, white wine, port, spirits and other) they consumed per week. These were converted to grams and coded as whether the participant exceeded the UK Chief Medical Officer guidelines of 14 units (112g) per week [37]. Body Mass index (BMI) was calculated from height and weight measurements taken at the initial Assessment Centre visit, and categorised as ‘Underweight’. ‘Healthy weight’, ‘Overweight’ and ‘Obese’ based on WHO cut-offs [28]. For analysis we combined underweight (<1%) with those of healthy weight into a single category ‘Not overweight or obese’. As the BMI measurement preceded the accelerometer measurement, we treated it as a confounder, although in general the relationship may be bi-directional[29].

Calculation of Hazard Ratios for intensity distribution

Interpreting the intensity distribution smooth function is complex and depends on the comparison of different PA intensity distributions. We constructed approximate Hazard Ratios (HRs) and 95% credible intervals to compare high-risk, low-risk and medium-risk intensity distributions (risk profiles) as follows:

First, we estimated the contribution of PA volume and intensity distribution to the modelled outcome for each participant as the predicted value from the model, controlling for all other covariates. We divided participants into groups based on twenty quantiles (ventiles) of PA volume, and classified each participant as having a high-, average- or low-risk intensity profile depending on whether they had high, average, or low predicted values of risk within that ventile. A high-risk profile was defined as having a predicted value higher than the 75th percentile for that ventile, a low risk profile below the 25th percentile and medium risk in between. Thus, a high-risk profile is one with an estimated higher risk compared to other participants with a similar PA volume. Finally, we used a simulation-based approach[30, p293ff] to estimate the hazard from the model for high- and low-risk profiles separately and computed HRs and 95% credible intervals via summaries of the simulations.

We used a similar approach to produce the plot in Figure 3, by predicting mortality risk and categorising into twenty intensity profile risk groups, within each PA volume quartile. HRs were calculated compared to a participant with average PA volume and a medium risk profile and plotted against risk profile for different levels of mean PA volume.

List of Figures

Figure S1: Mean histogram of accelerometer intensity: bars represent the average density in each bin across all participants.

Figure S2: Associations between physical activity volume and mortality: full range of data for mean accelerometer intensity

List of Tables

Table S1: Change in confounders between recruitment and T1 (includes those not in accelerometer subsample) N=20,264

Table S2: Hazard ratios for the association between physical activity volume and intensity histogram and mortality (excluding COVID pandemic)

Table S3: Model fit for more complex models

Figure S1: Mean histogram of accelerometer intensity: bars represent the average density in each bin across all participants, with shaded bars indicating 95% intervals.
Intensities 200-2000m*g* not shown (account for <3%).

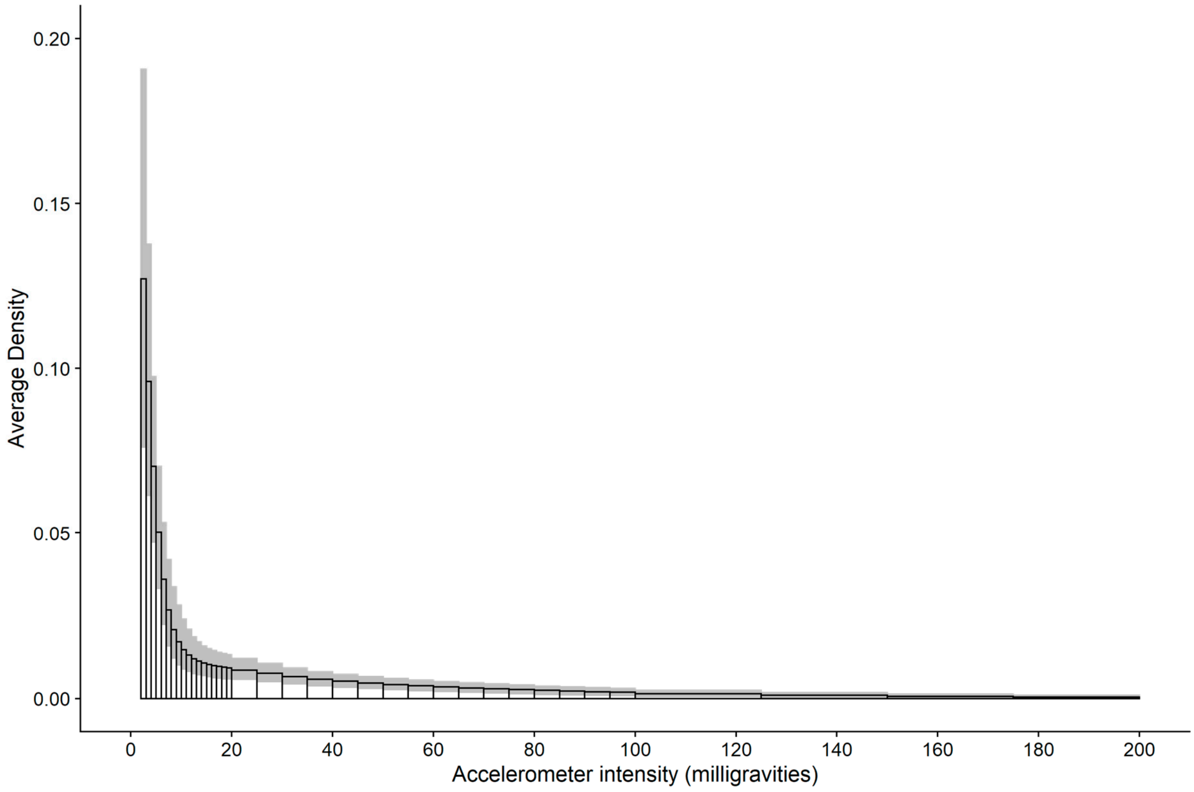

Figure S2: Associations between physical activity volume and mortality: full range of data for mean accelerometer intensity

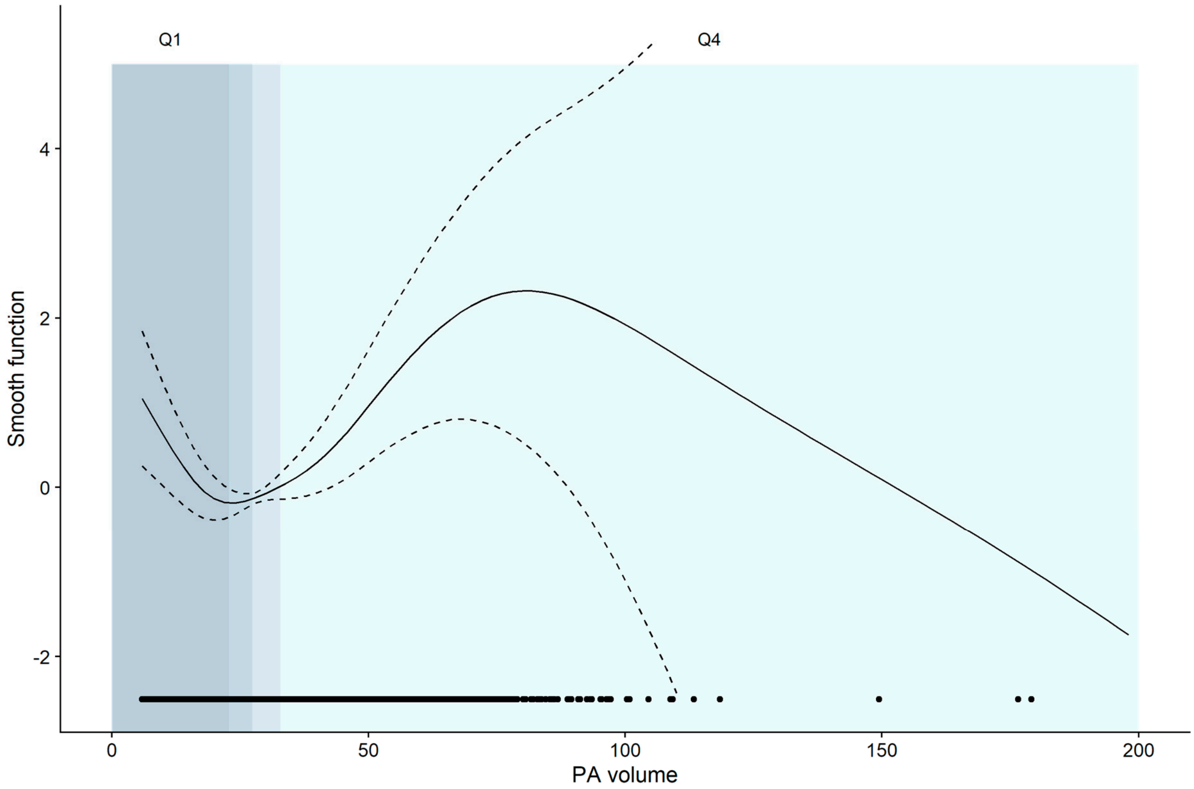

Shaded areas indicate quartiles of the physical activity volume (mean intensity), and dots along the x axis indicate cases. The ‘bump’ between 70mg and 100mg is due to five highly influential outliers.

Table S1: Change in confounders between recruitment and T1[a] (n=20 264: includes those with and without accelerometer data)

	Percentage with same value at both time points
Degree-educated	92%
Self-reported poor health	97%
Long-standing illness	83%
Current smoker	97%
Exceeds alcohol guidelines	87%
BMI category	82%

[a] T1 was between August 2012 and June 2013

Table S2: Hazard ratios for the association between physical activity volume and intensity histogram and all-cause mortality (end point of 1st March 2020: excluding COVID pandemic)

	HR[d]	95% CI
Male (vs female)	1.67	(1.49, 1.89)
Age (10-year increase)	2.45	(2.24, 2.68)
Degree-educated	0.89	(0.80, 1.00)
Townsend index (1 sd[a] increase)	1.02	(1.00, 1.04)
Self-reported poor health	1.83	(1.42, 2.35)
Long-standing illness	1.39	(1.24, 1.56)
Current smoker	1.88	(1.59, 2.23)
Exceeds alcohol guidelines	1.07	(0.95, 1.20)
Overweight[b]	0.94	(0.83, 1.07)
Obese[b]	1.16	(0.99, 1.34)
Mean PA volume (increase of 1m*g*)[c]		
0 – 20m*g*	0.90	(0.85, 0.94)
20 – 30m*g*	0.99	(0.96, 1.03)
30 – 80m*g*	1.05	(1.01, 1.09)
80m*g*+	0.98	(0.88, 1.10)
PA intensity histogram:		
Move from high risk to average risk	0.83	(0.78, 0.88)
Move from average risk to low risk	0.79	(0.73, 0.87)
Move from high risk to low risk	0.66	(0.57, 0.77)

HR= hazard ratio; CI = confidence interval; sd = standard deviation; PA = physical activity; m*g* = milligravities

[a] Townsend standard deviation = 2.8

[b] Compared to ‘not overweight or obese’

[c] Refers to the mean physical activity volume (accelerometer mean intensity). An increase of 1mg is roughly equivalent to replacing 10 minutes of sedentary time with 10 minutes of moderate walking.

[d] Adjusted for sex, age, education, Townsend deprivation, self-reported poor health, long-term illness, current smoking, exceeding alcohol guidelines, and BMI category.

Table S3: Model fit for more complex models

	AIC[a]
Main model	38561.71
nonlinear association with age	38563.66
Intensity distributions by sex	38566.21

[a] Akaike Information Criterion

AIC is a measure of model fit, with lower values indicating better fit. As a general rule of thumb, a difference in AIC of less than 5 provides no support to favour one model over another.
