# Supplementary material for: Tortoise or Hare? The Associations between Physical Activity Volume and Intensity Distribution and the Risk of All-Cause Mortality: A Large Prospective Analysis of the UK Biobank
Source: Int J Environ Res Public Health. 2023 Jul 19;20(14):6401. doi: 10.3390/ijerph20146401 (PMC10378963; doi:10.3390/ijerph20146401)
Supplement: Supplementary file 1 [file ijerph-20-06401-s001.zip › Technical Supplement S2.pdf]

Supplementary on methods and sample R code

Models

For investigating the relationship between mortality (Model 1), physical activity and other covariates we use a smooth additive Cox model [3] which is an extension of the Cox model [2]. For parameter estimation including smoothness parameters we use a penalised version of the partial likelihood of the smooth additive Cox model, as implemented in the gam function of the mgcv package [5, 4] in R. We model the survival time t_i of individual i with time t_i equal to the time from the accelerometer measurement.

This model relates the hazard function, $h(t)$, to the baseline hazard function, $h_0(t)$. A hazard function is a measure of risk or *hazard* of some event occurring, in our case it is the risk of death. The baseline hazard function is the underlying hazard common to all individuals in the population. All of our covariates are invariant in time, hence the following smooth additive Cox model for the hazard is used. Note this is an extension of the model used in [1].

$$h(t_i) = h_0(t_i) \exp \left\{ \mathbf{x_i}^\mathsf{T} \boldsymbol{\beta} + \sum_j f_1(p_j) z_i(p_j) + \gamma \texttt{accel}_i + \mathbf{w}^\mathsf{T} \mathbf{b} \right\}.$$

The vector $\mathbf{x_i}$ contains the variables male, age, degree-educated, Townsend index, self-reported poor health, long-standing illness, current smoker, exceeds alcohol guidelines, overweight (not overweight, overweight, obese) and $\boldsymbol{\beta}$ are the associated coefficients. The vector $\mathbf{w}$ is a factor for assessment centre and $\mathbf{b}$ is the associated random effect. The variable `accel` is mean intensity of physical activity (in milligravities mg per 5 seconds), and the functional variable $z_i(p_j)$ is the relative frequency of the histogram with some given number at mid-point p_j of physical activity intensity. The f_1 is a thin plate regression spline where we exclude the penalty null space. This is useful for testing whether the smooth function could be replaced by a linear term. In this particular context we use it to test whether it is necessary take the to intensity distribution into account in addition to mean intensity, because the linear part of the smooth applied to the relative frequencies of the histogram is equal to $\gamma \sum_j p_j z_i(p_j) = \gamma \texttt{accel}_i$.

In a second step we allow the effect of `accel` to be nonlinear using a thin plate regression spline (f_2)

$$h(t_i) = h_0(t_i) \exp \left\{ \mathbf{x_i}^\mathsf{T} \boldsymbol{\beta} + \sum_j f_1(p_j) z_i(p_j) + f_2(\texttt{accel}_i) + \mathbf{w}^\mathsf{T} \mathbf{b} \right\}.$$

References

[1] Augustin, N. H., Mattocks, C., Faraway, J. J., Greven, S., and Ness, A. R. (2017). Modelling a response as a function of high-frequency count data: The association between physical activity and fat mass. *Statistical methods in medical research*, 26(5):2210–2226.

[2] Cox, D. R. (1972). Regression models and life-tables. *Journal of the Royal Statistical Society. Series B (Methodological)*, 34(2):187–220.

[3] Hastie, T. and Tibshirani, R. (1987). Generalized additive models: some applications. *Journal of the American Statistical Association*, 82(398):371–386.

[4] Wood, S. (2017). *Generalized Additive Models: An Introduction with R*. Chapman and Hall/CRC, 2 edition.

[5] Wood, S., N., Pya, and Säfken, B. (2016). Smoothing parameter and model selection for general smooth models (with discussion). *Journal of the American Statistical Association*, 111:1548–1575.

R code

```
#  model 1:linear mean intensity plus histogram:
mod.1=gam(time ~s(centre,bs="re")+
               male + age +
               degree + townsend +
               poor.health + illness +
               current.smoker + alcohol + overwt + obese +
               accel+
               s(midptdata,k=30,by=prop,bs="tp",m=c(2,0)),
# m=c(2,0) specifies a thin plate regression spline with a second order penality
# and no null space  (no linear part in basis), see p. 313 in Wood, SN (2017)
             family=cox.ph(), data=biobank, weights=death, method="REML")

summary(mod.full)
AIC(mod.full)
BIC(mod.full)


# Full model: nonlinear mean intensity plus histogram:
mod.full=gam(time ~s(centre,bs="re")+
               male + age +
               degree + townsend +
               poor.health + illness +
               current.smoker + alcohol + overwt + obese +
               s(accel,bs="tp")+
               s(midptdata,k=30,by=prop,bs="tp",m=c(2,0)),
             family=cox.ph(), data=biobank, weights=death, method="REML")

summary(mod.full)
AIC(mod.full)
BIC(mod.full)


################################################################
# Hazard Ratios
################################################################


## Get data and drop missings

biobank.cc=na.omit(biobank[,c("id","centre","male","age","degree","townsend",
                              "poor.health","illness","current.smoker",
                              "alcohol","overwt","obese","accel")])
bio.cols=3:13
cc=biobank$id %in% biobank.cc$id


################### Function to create a list with zero elements
################### for predictions/ses etc

default.newdata.fn=function(data, cols=bio.cols,elements="zeros",npred=1,centre=11011,mid=midpt,p=prop) {

  nc=length(cols)
  if (elements=="means") {
    tmp=apply(data[,cols],2,mean,na.rm=T)
  }
```

```
  else {
    tmp=rep(0,nc)
    names(tmp)=names(data[,cols])
  }

  newdata=vector("list",nc)
  names(newdata)=names(tmp)
  for (i in 1:nc) {
    newdata[[i]]=rep(tmp[i],npred)
  }

  newdata$midptdata=matrix(rep(mid,npred),npred,length(mid),byrow=T)
  newdata$prop=matrix(rep(apply(p,2,mean,na.rm=T),npred),npred,dim(p)[2],byrow=T)

  newdata$centre=matrix(rep(centre,npred),npred,1)
  newdata$centre=factor(newdata$centre)

  newdata
}


##################################
#HR for parametric terms
##################################

change=c(1,10,1,1,1,1,1,1,1,1)
hr.full= matrix(exp(matrix(change,10,3)*
                      (coef(mod.full)[1:10] +
                         rep(c(0,-1,1),rep(10,3))*
                         1.96*summary((mod.full))$se[1:10])),10,3)

hr.full=as.data.frame(hr.full)
names(hr.full)=c("HR","lower 95%","upper 95%")
hr.full$var=names(coef(mod.full)[1:10])
hr.full$change=change
hr.full

##################################
#HR for mean PA volume
##################################

newdata=default.newdata.fn(biobank.cc,npred=5)

# Compare parts of the curve: scale to an increase in 1mg
newdata$accel=c(0,20,30,80,100)
Xp=predict(mod.full,newdata,type="lpmatrix")
fv=Xp %*% coef(mod.full)
v=vcov(mod.full)
#0-20
d=t(c(-1,1,0,0,0))/20
exp(cbind(d %*% fv,sqrt(d %*% Xp %*% v%*% t(Xp) %*% t(d))) %*%
      matrix(c(1,0,1,-1.96,1,1.96),nrow=2))
#20-30
d=t(c(0,-1,1,0,0))/10
exp(cbind(d %*% fv,sqrt(d %*% Xp %*% v %*% t(Xp) %*% t(d))) %*%
      matrix(c(1,0,1,-1.96,1,1.96),nrow=2))

#30-80
d=t(c(0,0,-1,1,0))/50
exp(cbind(d %*% fv,sqrt(d %*% Xp %*% v %*% t(Xp) %*% t(d))) %*%
      matrix(c(1,0,1,-1.96,1,1.96),nrow=2))

#80+
d=t(c(0,0,0,-1,1))/20
exp(cbind(d %*% fv,sqrt(d %*% Xp %*% v %*% t(Xp) %*% t(d))) %*%
      matrix(c(1,0,1,-1.96,1,1.96),nrow=2))

#In the very low PA volume group (0-20) increasing by 2mg:
d=t(c(-1,1,0,0,0))/10
exp(cbind(d %*% fv,sqrt(d %*% Xp %*% v%*% t(Xp) %*% t(d))) %*%
      matrix(c(1,0,1,-1.96,1,1.96),nrow=2))
# is roughly equivalent to the benefits of switching from
# high risk to average risk intensity profile
# 0-20mg inc by 2mg:     0.84 (0.76, 0.91)
# high risk -> low risk: 0.83 (0.79, 0.88)

##################################
# HRs for intensity distribution
##################################

newdata=default.newdata.fn(biobank.cc,cols=bio.cols,npred=nrow(biobank.cc))

# populate with actual accel and distn
newdata$prop=prop[which(biobank$id %in% biobank.cc$id),]
newdata$accel=biobank.cc$accel


#predict and simulate from predictive distn
set.seed(2108)
beta.sim=rmvn(n=1000,coef(mod.full),vcov(mod.full))
Xp=predict(mod.full,newdata,type="lpmatrix",unconditional=T)

# Classify into accel groups based on 5-percentiles
```

```
p5=quantile(biobank.cc$accel,p=seq(0,1,by=0.05))
#set max and min a little bigger to include everyone
p5[1]=0
p5[21]=p5[21]+10

biobank.cc$p5=cut(biobank.cc$accel,p5,labels=paste("ptile ",(0:19)*5,"-",(1:20)*5,sep=""))
table(biobank.cc$p5)


#Calculate fitted value
biobank.cc$pred=Xp %*% coef(mod.full)

# Classify profiles into high, med and low risk
# depending on accel group
#   high risk: pred is above 75th ptile for that accel group
#   med risk: pred is between 25-75th ptile for that accel group
#   low risk: pred is below 25th ptile for that accel group

biobank.cc$risk=2
for (p in 1:20) {
  # select those in the current ptile group
  gp=(as.numeric(biobank.cc$p5)==p)
  # calculate ptiles
  p25=quantile(biobank.cc$pred[gp],0.25)
  p75=quantile(biobank.cc$pred[gp],0.75)
  # assign risk group: 1=low 2=med 3=high
  biobank.cc$risk[gp][biobank.cc$pred[gp]<p25]=1
  biobank.cc$risk[gp][biobank.cc$pred[gp]>p75]=3
}

table(biobank.cc$risk)
summary(biobank.cc$pred[biobank.cc$risk==1])
summary(biobank.cc$pred[biobank.cc$risk==2])
summary(biobank.cc$pred[biobank.cc$risk==3])

#hi ->mid
d1=matrix(0,1,nrow(biobank.cc))
d1[biobank.cc$risk==3]=-1/sum(biobank.cc$risk==3)
d1[biobank.cc$risk==2]=1/sum(biobank.cc$risk==2)

#mid ->lo
d2=matrix(0,1,nrow(biobank.cc))
d2[biobank.cc$risk==1]=1/sum(biobank.cc$risk==1)
d2[biobank.cc$risk==2]=-1/sum(biobank.cc$risk==2)

#hi -> lo
d3=matrix(0,1,nrow(biobank.cc))
d3[biobank.cc$risk==3]=-1/sum(biobank.cc$risk==3)
d3[biobank.cc$risk==1]=1/sum(biobank.cc$risk==1)

#HR with CI
quantile(exp(d1 %*% Xp %*% t(beta.sim)),p=c(.5,.025,.975))
quantile(exp(d2 %*% Xp %*% t(beta.sim)),p=c(.5,.025,.975))
quantile(exp(d3 %*% Xp %*% t(beta.sim)),p=c(.5,.025,.975))
```
